# AN EXAMINATION OF ADVANCED CARE PLANNING IN BLACK AMERICANS: DIRECTION TO IMPROVE ENGAGEMENT

**DOI:** 10.1093/geroni/igac059.3083

**Published:** 2022-12-20

**Authors:** Rachel Bassett, Daniel Paulson, Nicole Valdes McClure

**Affiliations:** University of Central Florida, Orlando, Florida, United States; University of Central Florida, Orlando, Florida, United States; University of Central Florida, Orlando, Florida, United States

## Abstract

Past studies have found that older Black Americans are less likely to establish advance care directives (ACDs) than their White age peers, and medical distrust has been identified as one possible cause. Others have suggested the formulaic approach of ACD documentation which may conflict with the spiritual and cultural nature of these decisions. The Five Wishes ACD was developed partially to address this need. This study seeks to 1) replicate past findings regarding race and ACD adoption, and test the hypotheses that 2) Race will differentially predict Advanced Care Planning (ACP) engagement scores between conditions (Five Wishes vs State Directive), and 3) level of trust in physicians will relate to ACP engagement. The sample (N = 186) was recruited from Amazon Mechanical Turk and Prolific. Participants ranged from 50 to 77 years, were predominantly female (56.8%), White (51.61%), married (52.6%), and college-educated (70 .6%). ANCOVA results were a nonsignificant main effect of ACP engagement by race (F(1, 185) = 1.93, p = .166) and nonsignificant interaction of race by condition (F(1, 185) = 0.16, p < .69). Trust in physician scale scores predicted ACP engagement (F(1, 185) = 16.15, p < .001). The lack of an effect of race on ACP utilization may be explained by educational and SES characteristics of the sample by contrast to prior studies. The Five Wishes and State Directive ACD documents resulted in similar ACD engagement. These results suggest that trust in physicians is a primary barrier to ACP utilization.

